# Nitrogen-doped twisted graphene grown on copper by atmospheric pressure CVD from a decane precursor

**DOI:** 10.3762/bjnano.8.15

**Published:** 2017-01-16

**Authors:** Ivan V Komissarov, Nikolai G Kovalchuk, Vladimir A Labunov, Ksenia V Girel, Olga V Korolik, Mikhail S Tivanov, Algirdas Lazauskas, Mindaugas Andrulevičius, Tomas Tamulevičius, Viktoras Grigaliūnas, Šarunas Meškinis, Sigitas Tamulevičius, Serghej L Prischepa

**Affiliations:** 1 Belarusian State University of Informatics and Radioelectronics, P. Brovka str. 6, Minsk 220013, Belarus; 2 Belarusian State University, Nezavisimosti av. 4, Minsk 220030, Belarus; 3 Institute of Materials Science, Kaunas University of Technology, K. Baršausko str. 59, Kaunas 51423, Lithuania

**Keywords:** G-resonance, nitrogen doping of graphene, Raman spectroscopy, twisted graphene, X-ray photoemission spectroscopy

## Abstract

We present Raman studies of graphene films grown on copper foil by atmospheric pressure CVD with n-decane as a precursor, a mixture of nitrogen and hydrogen as the carrier gas, under different hydrogen flow rates. A novel approach for the processing of the Raman spectroscopy data was employed. It was found that in particular cases, the various parameters of the Raman spectra can be assigned to fractions of the films with different thicknesses. In particular, such quantities as the full width at half maximum of the 2D peak and the position of the 2D graphene band were successfully applied for the elaborated approach. Both the G- and 2D-band positions of single layer fractions were blue-shifted, which could be associated with the nitrogen doping of studied films. The XPS study revealed the characteristics of incorporated nitrogen, which was found to have a binding energy around 402 eV. Moreover, based on the statistical analysis of spectral parameters and the observation of a G-resonance, the twisted nature of the double-layer fraction of graphene grown with a lower hydrogen feeding rate was demonstrated. The impact of the varied hydrogen flow rate on the structural properties of graphene and the nitrogen concentration is also discussed.

## Introduction

Single layer graphene (SLG) exhibits exceptional electronic properties, making it one of the most advanced materials of our time. Due to its high charge carrier mobility [1], it has huge functional ability in many applications, especially in high frequency electronics. The increase in the number of layers with conventional Bernal stacking strongly affects the electronic properties of graphene. Contrary to monolayer graphene, in Bernal-stacked graphene multilayers, electron backscattering is allowed [2]. However, when the layers of graphene are not strongly electronically coupled, this scenario is not always realized. Indeed, for turbostratic graphite, where Bernal stacking is destroyed, even for a very large number of layers, the unique properties of graphene can be preserved [3]. Double (triple) layer turbostratic graphite is also known as twisted graphene (TG). This term reflects the fact that both the electronic and structural properties of double layer graphene can be well-described by the in-plane rotation angle θ between the graphene layers. Many theoretical and experimental studies have focused on the unique properties of TG [4]. In particular, it was demonstrated that for angles θ *>* 10°, the layers are electronically decoupled, and the low-energy band structure looks like a simple superposition of the Dirac cones of the individual graphene planes [5–6]. For SLG, the Fermi velocity reaches the value 10^6^ m/s for θ *>* 10° and drastically decreases for θ *<* 5° [5]. In addition, one of the most attractive characteristics of TG is the pair of logarithmic divergences in the density of states, known as the van Hove singularities (vHs), which are formed due to the overlap of the Dirac cones in the *k*-space.

The controlled injection of defects, which cause strain, is an extra degree of freedom in addition to the number of layers. This may account for the unique properties of TG. In particular, it was shown that the strained TG bilayer could be an ideal platform for the realization of the high-temperature zero-field quantum valley Hall effect [7]. From a practical point of view, the band gap opening in the electronic structure of graphene is quite attractive. It is expected that this will result in a new approach for application of graphene in digital electronics. It was also theoretically predicted that, in TG with small uniaxial strain (which comprises only a few percent), a finite conduction gap as large as hundreds of meV can be obtained [8]. Thus, the study of the defect impact on the TG properties is quite a motivating topic from both fundamental and applied aspects.

TG can be obtained by different methods, e.g., by means of graphene folding, graphene layer stacking, thermal decomposition of SiC [9] or chemical vapor deposition (CVD) on metal catalysts [10–11]. Generally speaking, CVD is one of the most common methods to obtain large area and high quality graphene [12]. Moreover, TG may be grown at ambient pressure applying atmospheric pressure CVD (APCVD) [10]. The use of different hydrocarbon sources to explore the growth mechanism and properties of TG is a hot topic nowadays. Generally, methane (CH_4_) is the most common hydrocarbon used in the CVD process to grow graphene. However, the use of hydrocarbons other than CH_4_ compounds is a challenging task. The successful implementation may offer the possibility to tune the growth process and to pave the way to graphene synthesis with desirable parameters, such as type of defects and their concentration.

In this work, we investigate the experimental conditions at which the APCVD growth of large area, nitrogen-doped TG can be realized utilizing *n*-decane as a precursor in the presence of nitrogen flow.

## Experimental

### Synthesis and transfer

A custom-made APCVD set-up with a 14 mm diameter tubular quartz reactor was employed for the experiment. Polycrystalline copper foil (99.9% purity, proved by the EDX study) with a thickness of 60 μm was used as the catalyst. Prior to the APCVD, the foil was electrochemically polished for 5 min in 1 M phosphoric acid at a bias voltage of a 2.3 V. The 35 × 45 mm^2^ sample was placed in the middle of the reactor. One side of the copper foil covered the inner wall of the reactor. The copper foil was annealed for one hour at 1050 °C in the presence of N_2_ and H_2_ gas flow at a rate of 100 and 150 cm^3^/min, respectively. The purity of the nitrogen gas was 99.95%. We utilized a commercial hydrogen generator (GVCh-12D) as a H_2_ source. The resulting purity of the hydrogen gas was 99.99%. The temperature was controlled by a thermocouple placed inside the heating block, next to the reactor wall.

The above experimental procedure has been previously reported in [13–14]. The key differences in the current experimental approach are related to the position of the copper foil in the reactor, the thickness of the copper and the feed rate of *n*-decane. In addition, in previous publications [13–14], the aspect of nitrogen doping of graphene has not been investigated.

In this article, we present the results related to two samples: sample A and sample B. Sample A was prepared at 1050 °C in the presence of N_2_ and H_2_ gas flow with the rates of 100 and 60 cm^3^/min, respectively. Sample B was prepared under similar conditions, except the H_2_ gas flow rate was reduced to 6 cm^3^/min.

The APCVD depends essentially on the hydrocarbon precursor [15]. To this end, a precursor that has similar chemical properties but different molecular mass is desirable for a deeper understanding of the graphene growth kinetics. One of such candidates is *n*-decane (C_10_H_22_), a member of the homologous series of alkane hydrocarbons. *n*-Decane has a molecular mass approximately one order of magnitude greater than that of methane, which influences the growth kinetics of graphene. As a representative *n*-alkane, *n*-decane forms chains of radicals with a high reactive nature during thermal decomposition. This could stimulate numerous chemical reaction pathways and promote doping. In fact, CVD makes it possible to dope graphene by nitrogen in situ, which not only tolerates the ground state of graphene via additional electrons but also introduces a strain to graphene because of the difference in ionic radii [16]. The radicals resulting from the decomposition of *n*-decane could lead to the decomposition of the nitrogen molecule, which in fact has one of the strongest binding energies. The resulting atomic nitrogen can be embedded into the graphene lattice.

The *n*-decane was introduced into the tubular quartz reactor via barbotage system for 30 min. The feeding rate of *n*-decane was estimated to be 4 μL/min (for both samples). Afterwards, the tubular quartz reactor was cooled at a rate of 50 °C/min in the presence of N_2_ gas flow. The obtained properties of samples A and B are typical for samples synthesized under similar conditions.

The transfer of graphene from the original to the arbitrary substrate without deteriorating the crystallinity of the graphene is still a challenging task [17]. Currently, there are two main approaches for the transfer of graphene. The first one consists of mechanical exfoliation, which imposes severe mechanical, uncontrolled defects in the sample. The most common and preferable is the wet-chemical etching of the catalyst (substrate). Usually a poly(methylmethacrylate) (PMMA) scaffold is applied to coat the graphene surface and support it during the catalyst consumption, followed by underside contaminant cleaning, then placement on the destination substrate. However, the PMMA removal from the graphene after the film transfer (which involves high-temperature Ar/H_2_ forming gas annealing [18], O_2_-based annealing [19], and in situ annealing [20]), deteriorates the graphene crystallinity. Additionally, these processes are operated at high temperatures, which restricts the application of graphene, including its use in flexible electronics and biomolecule encapsulation [21]. In this work, we employed a wet-chemical room temperature transfer process onto SiO_2_(598 nm)/Si substrates without the use of a polymer support. This was performed in two steps. First, one side (the side that was next to the reactor wall) of the copper foil was treated for 3 min in a solution of H_2_NO_3_ and H_2_O mixed in a volume ratio of 1:3, and then the copper foil was totally dissolved in a water solution of FeCl_3_. The graphene film was gently washed several times in a bath with distilled water prior to the transfer onto the substrate.

### Characterization

The graphene samples were analyzed by Raman spectroscopy using the Nanofinder HE with 532 nm and 473 nm excitation wavelengths and a Confotec NR500 confocal micro-Raman spectrometer with 473 nm excitation wavelength. The spectral resolution was about 3 cm^−1^ for both spectrometers. A 3D scanning laser confocal Raman microscope (Confotec NR500) allowed for the acquisition of two kinds of images within a single scan: a Rayleigh image, using laser light reflected from a sample, and a spectral image by Raman scattering. More details about the laser beam size and spectra accumulation time are presented further in the text.

A Thermo Scientific ESCALAB 250Xi spectrometer with monochromatic Al Kα radiation (*h*ν = 1486.6 eV) was used for X-ray photoelectron spectroscopy (XPS) measurements. The base pressure in the analytical chamber was lower than 2 × 10^−7^ Pa. The 20 eV and 40 eV pass energy values of a hemispherical electron energy analyzer were used for the survey and high resolution spectra acquisition, respectively. The energy scale of the system was calibrated with respect to Au 4f_7/2_, Ag 3d_5/2_ and Cu 2p_3/2_ peak positions. ESCALAB 250Xi Avantage software was used for the peak deconvolution and fitting procedure using a sum of Lorentzian–Gaussian (70:30) functions. The samples were analyzed as received and no surface cleaning procedure was applied. Finally, the transmittance was measured using the PROSCAN MC-121 spectrometer.

## Results

**Figure 1:**
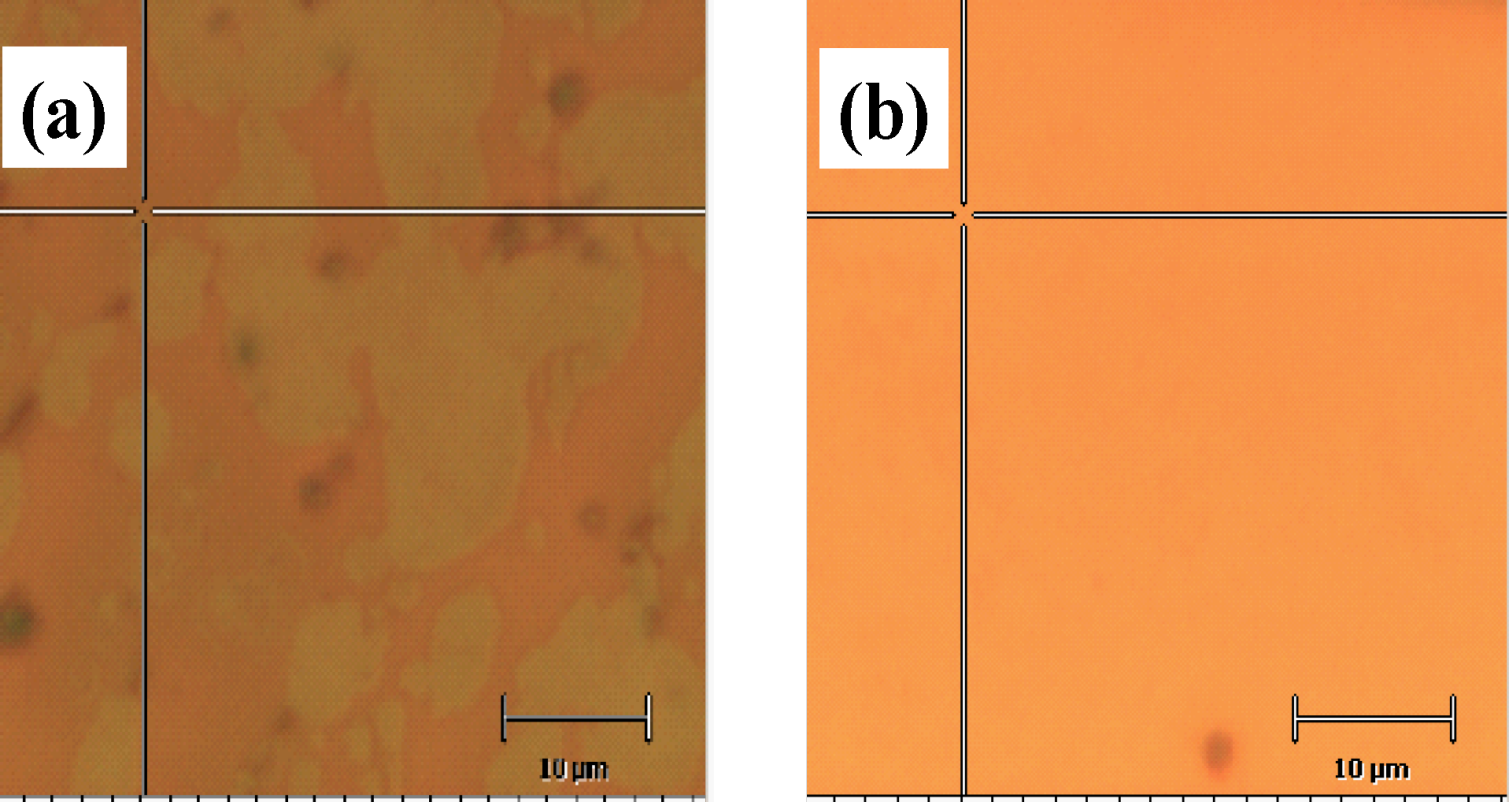
Optical images for (a) sample A, (b) sample B as deposited on copper foil.

The optical images of samples A and B on copper foil are shown in Figure 1a and Figure 1b, respectively. The surface of sample A contains randomly distributed hexagonally shaped spots (brighter areas), while the surface of sample B looks relatively homogeneous.

**Figure 2:**
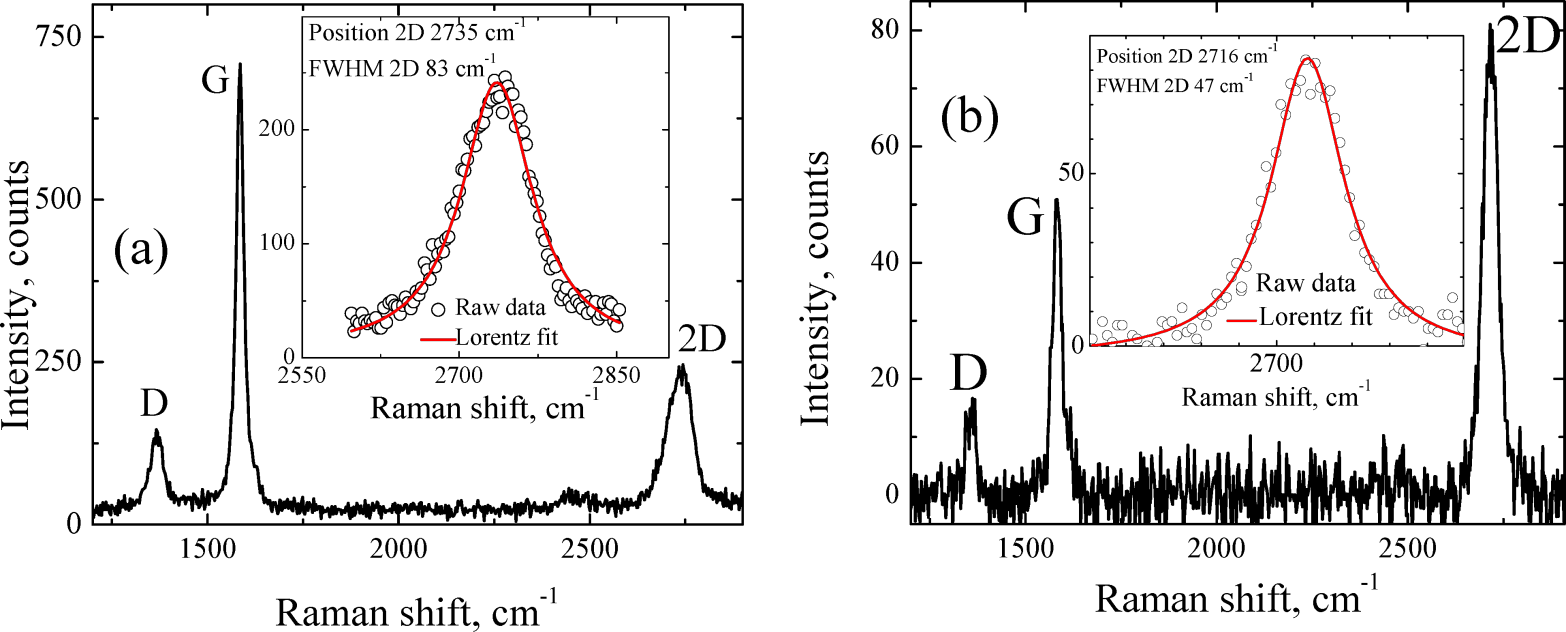
Raman spectrum for (a) sample A, (b) sample B on copper foil. The luminescence background from copper is subtracted. Insets: Measured 2D peak (symbols) with the Lorentz fit (line).

More information about the quality of the samples can be obtained from the Raman investigations. The resonance nature of Raman spectra in graphene makes them a versatile tool for studying both structural and electronic properties [22]. In Figure 2 we show the individual Raman spectrum of sample A acquired in the darker part, see Figure 1a. Figure 2b presents the Raman spectrum characteristic of sample B. A single spectrum was accumulated for 1 s with a laser excitation wavelength of 473 nm and a beam diameter of about 600 nm. In the insets to Figures Figure 2a and Figure 2b the 2D peaks are shown revealing the symmetry of the 2D band. The latter indicates the weak interlayer interaction, which will be discussed later.

**Figure 3:**
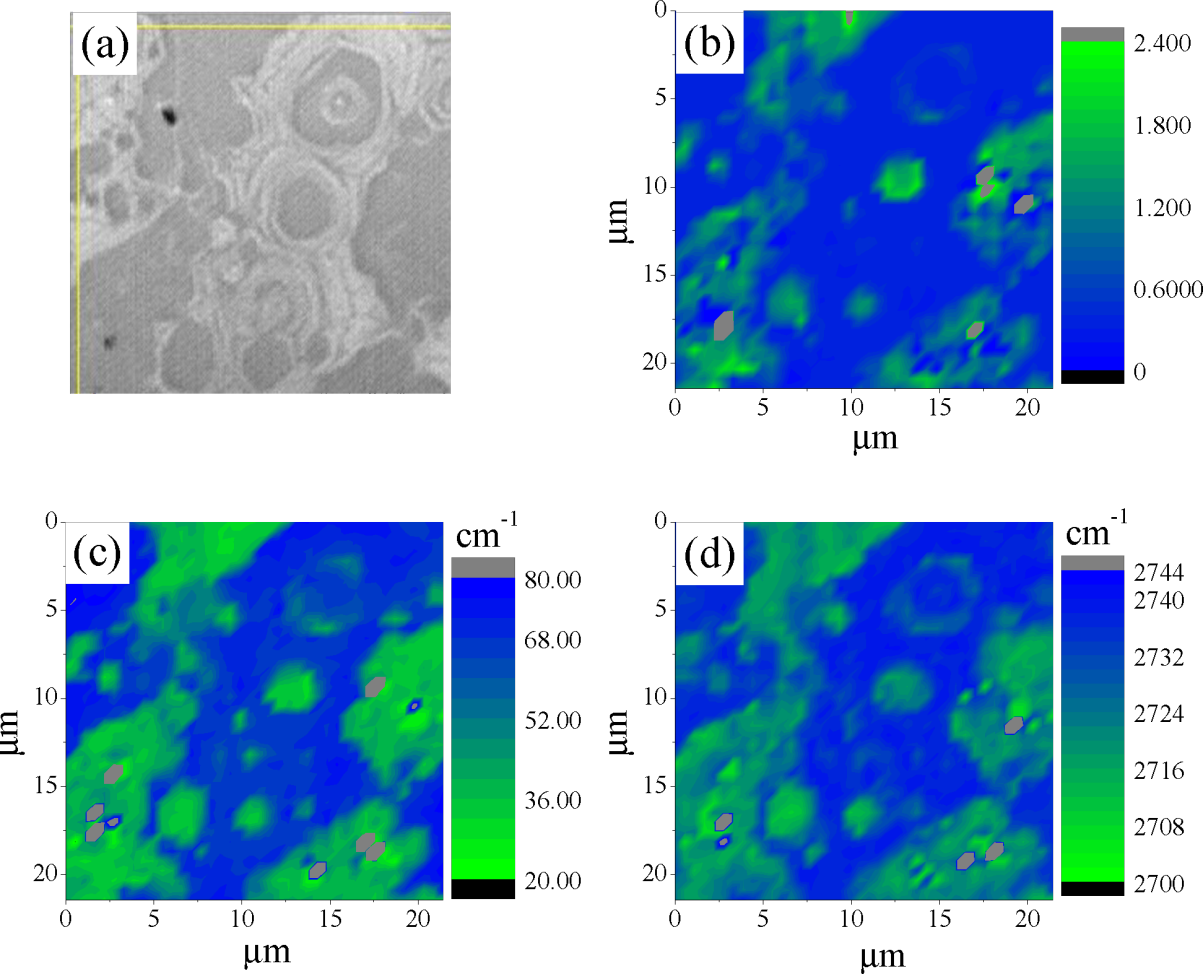
(a) The Rayleigh image. (b) *I*_2D_/*I*_G_ ratio map. (c) FWHM map of the 2D band. (d) 2D band position map. All data are for sample A on SiO_2_/Si substrate acquired with a laser excitation wavelength of 473 nm. Color coding represents the amplitude of measured values.

**Figure 4:**
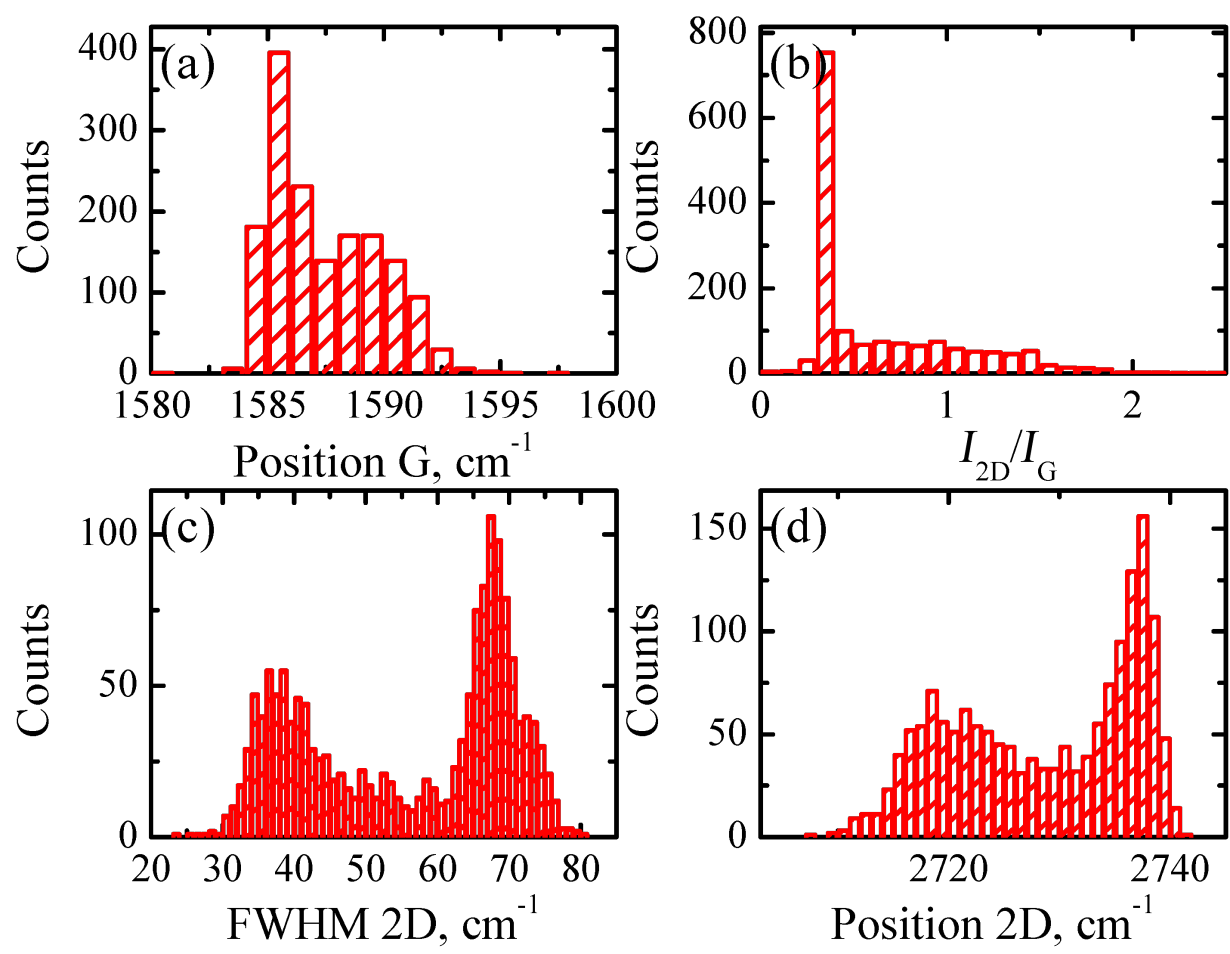
Raman mapping histograms for sample A on a SiO_2_/Si substrate as obtained from Raman maps of Figure 2. (a) G band position. (b) *I*_2D_/*I*_G_. (c) FWHM of the 2D band. (d) 2D band position.

In Figure 3a we demonstrate the Rayleigh image of sample A transferred to a SiO_2_/Si substrate. Raman mapping (1600 points) was performed for the same sample area as in Figure 3a. The results of this study are presented in Figure 3b–d and Figure 4, in which we show the relation between the intensities of the 2D and G bands, *I*_2D_/*I*_G_ (Figure 3b), full width at the half maximum (FWHM) map of the 2D band (Figure 3c), the 2D band map (Figure 3d) and corresponding Raman mapping histograms for the G band position (Figure 4a), *I*_2D_/*I*_G_ ratio (Figure 4b), FWHM of the 2D band (Figure 4c) and, finally, a histogram of the 2D band position (Figure 4d).

The correlation between the Rayleigh image (Figure 3a) and *I*_2D_/*I*_G_ ratio (Figure 3b) is clearly seen. The domains with *I*_2D_/*I*_G_ greater than 0.6 correspond to the dark spots in the optical image. The FWHM map of the 2D band (Figure 3c) and corresponding histogram (Figure 4c) suggest that a significant part of the sample is associated with the domains in which this parameter is less than 40 cm^−1^, which is typical for SLG [23]. However, the Raman map of the 2D band positions (Figure 3d) and corresponding histogram (Figure 4d) indicate that only a negligible part of the film surface is associated with values smaller than 2710 cm^−1^. It is important to note that the 2D band position of graphene transferred onto SiO_2_ and measured with the 473 nm laser excitation wavelength should be at 2703 cm^−1^ [24]. This means that the position of the 2D band is blue-shifted in sample A. The significant blue shift in the position is also observed for the G band (Figure 4a), 1585 cm^−1^ and 1588 cm^−1^ against 1580 cm^−1^ for SLG [25].

**Figure 5:**
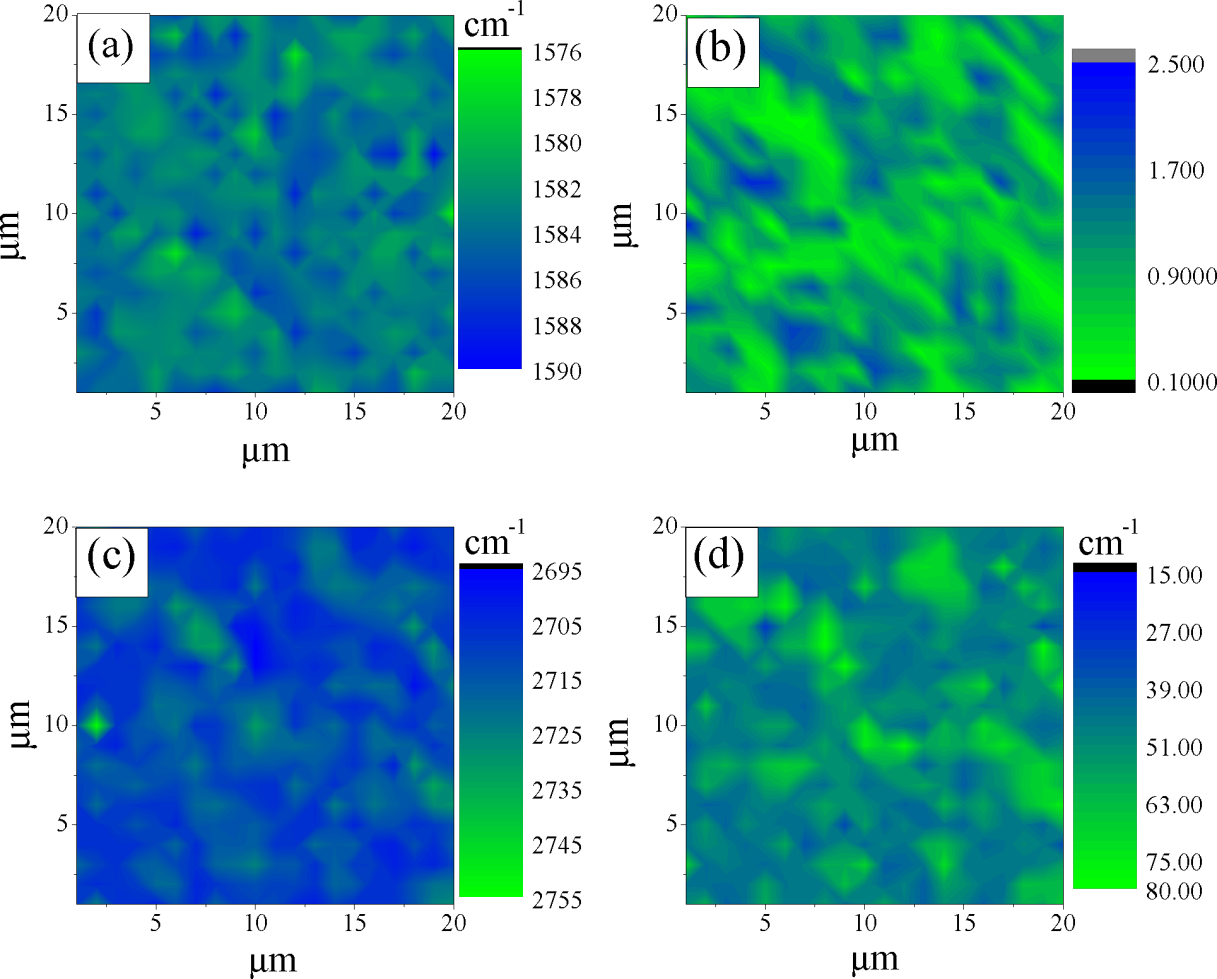
Raman maps of sample B on a SiO_2_/Si substrate acquired at a laser excitation wavelength of 473 nm. (a) G band position. (b) *I*_2D_/*I*_G_ ratio. (c) FWHM of the 2D band. (d) 2D band position.

**Figure 6:**
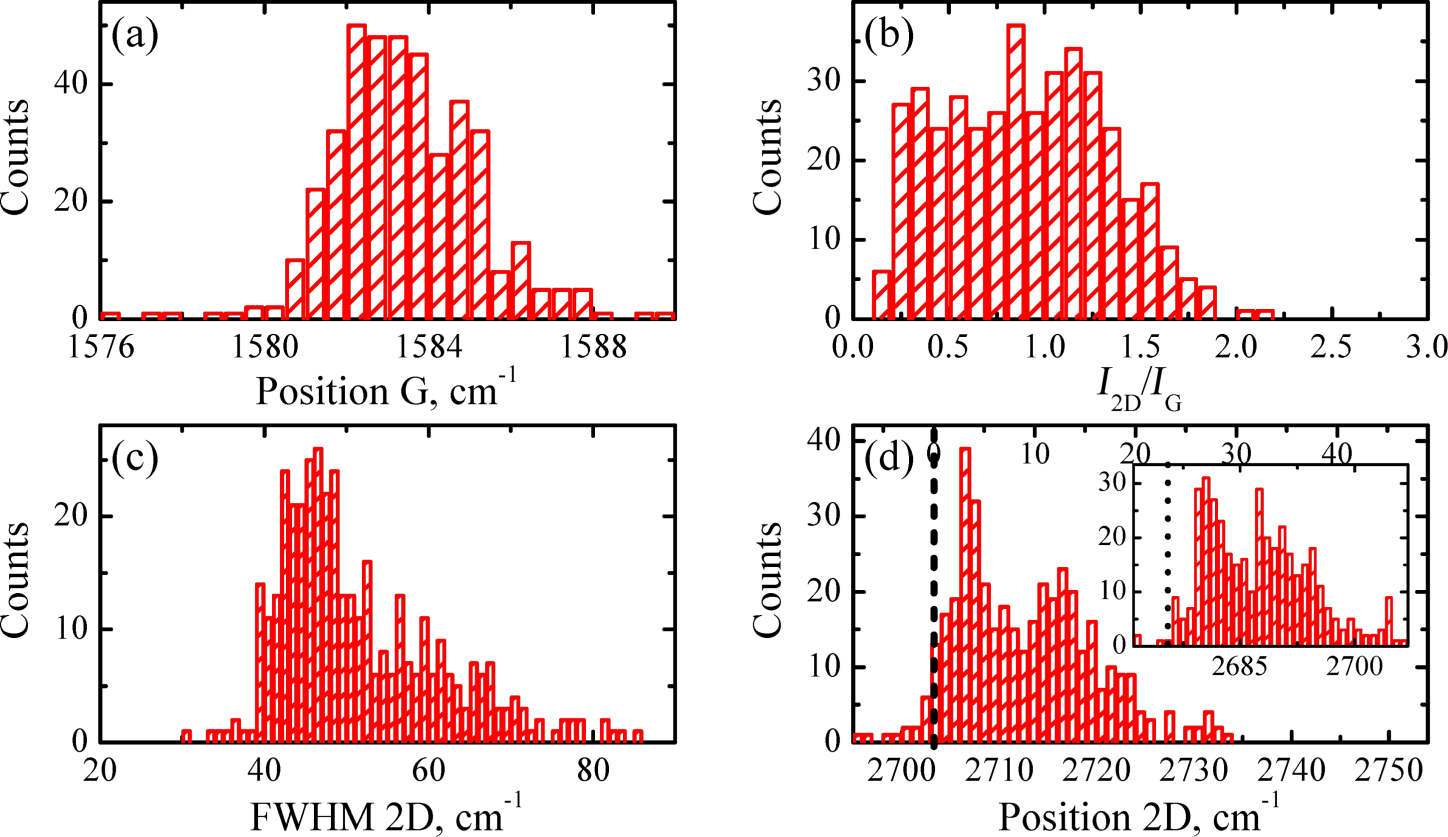
Raman mapping histograms for sample B on a SiO_2_/Si substrate as obtained from Raman maps of Figure 4. (a) G band position. (b) *I*_2D_/*I*_G_. (c) FWHM of the 2D band. (d) 2D band position. Inset: 2D band as measured for the laser excitation wavelength of 532 nm. The vertical dashed lines on panel (d) show the position of the 2D band of SLG on SiO_2_ according to [24].

Figure 5a–d shows Raman maps (400 points) of sample B transferred onto a SiO_2_/Si substrate. The corresponding histograms are presented in Figure 6a–d. A single Raman spectrum was accumulated for 10 s with a laser wavelength of 473 nm and a beam diameter of about 600 nm. The *I*_2D_/*I*_G_ ratio map (Figure 5b) shows the existence of domains with relatively uniform distribution. Moreover, the correlation between the *I*_2D_/*I*_G_ ratio (Figure 5b) and the 2D band position (Figure 5d) maps is observed.

The same area of sample B was analyzed with a different laser excitation wavelength (532 nm) utilizing the same laser beam conditions as for 473 nm excitation. By analogy with the previous Raman maps, the non-monotonic distribution of mapped values was observed. As an example, in the inset of Figure 6d we show the histogram for the 2D band position obtained with a 532 nm excitation wavelength. The similarity of the results for two different wavelengths is obvious.

## Discussion

### Partitioning of Raman data

In many aspects, the theory of Raman scattering in graphene is very well understood and the quantitative analysis of Raman spectra parameters provides sufficient information about structural and electrical properties of graphene. For example, the analysis of the line shape of the 2D band along with its spectral position can provide important information about the number of layers in graphene and the interlayer interaction [26]. From the G-band position, the carrier concentration can be obtained with high accuracy [27]. Finally, the analysis of the *I*_D_/*I*_G_ ratio is becoming a common method for the point defect concentration evaluation [28–29].

From Raman spectroscopy results presented in the current research it was found that a non-monotonic distribution was inherent for almost all data histograms presented in the Results section. For example, two sharp maxima are observed in the FWHM of the 2D band histogram of sample A (Figure 4c) at approximately 38 cm^−1^ and 67 cm^−1^, respectively. This suggests that within the same sample there are at least two graphene ”systems” with quite different numbers of layers. Indeed, the presence of two maxima in the 2D-position distribution (Figure 4d) is in good accordance with the observation of thicker and thinner domains in optical images (Figure 1a). Consequently, it is reasonable to analyze Raman spectra separately applying the criterion for data belonging to a particular peak. Following this idea, we split the Raman data of sample A into two sets, one of which contains data where the 2D FWHM values are smaller than 50 cm^−1^ and another is for data with the 2D FWHM greater than 50 cm^−1^.

**Figure 7:**
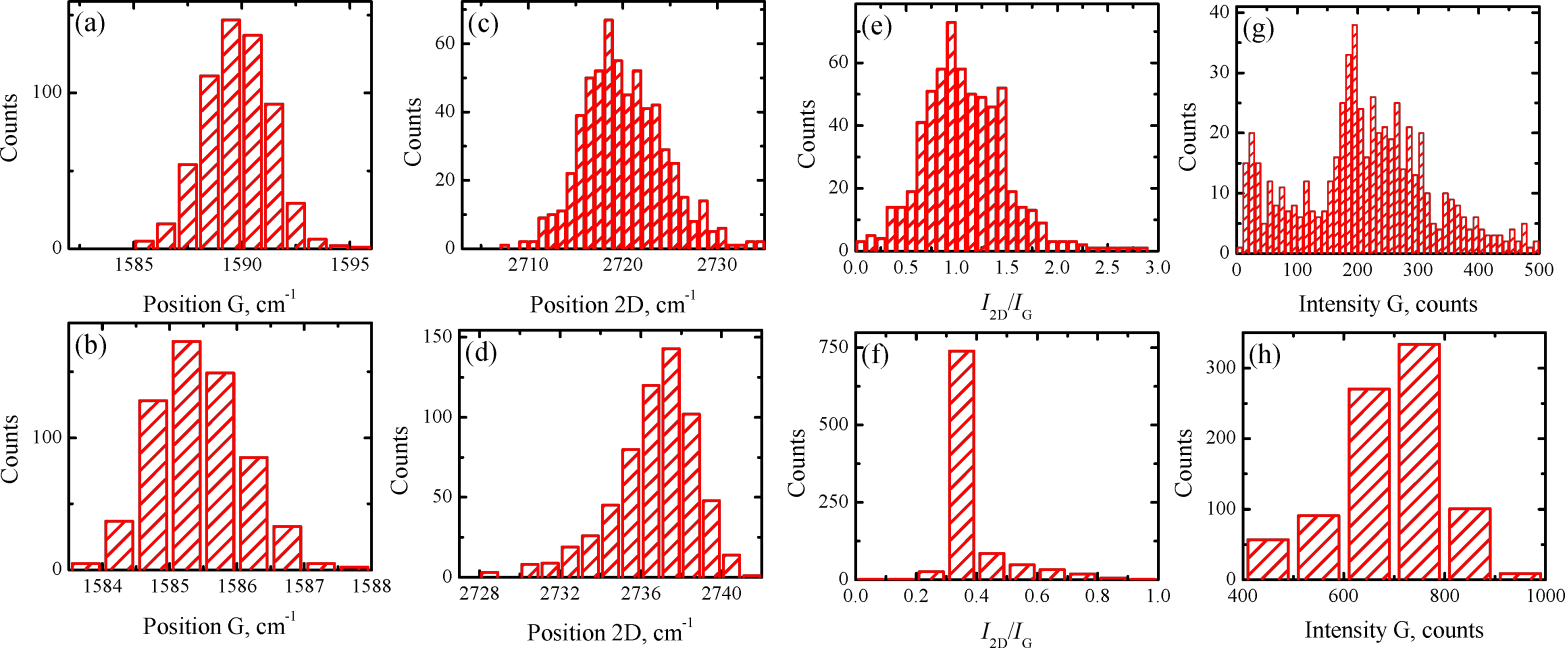
Replotted histograms of sample A on SiO_2_/Si substrate for laser excitation wavelength of 473 nm using 2D FWHM = 50 cm^−1^ as the partitioning criterion. (a) and (b) G-band position. (c) and (d) 2D-band position. (e) and (f) *I*_2D_/*I*_G_ ratio. (g) and (h) *I*_G_ value. Data shown in (a), (c), (e) and (g) plots are for 2D FWHM < 50 cm^−1^. Data shown in (b), (d), (f) and (h) plots are for 2D FWHM > 50 cm^−1^.

The histograms, replotted according to this criterion, are shown in Figure 7a–h. Data for the 2D peak FWHM smaller than 50 cm^−1^ are shown in Figure 7a,c,e,g, while data for the 2D peak FWHM greater than 50 cm^−1^ are presented in Figure 7b,d,f,h. It is clearly seen from Figure 7 that the histograms exhibit a relatively monotonic distribution with one distinct maximum. The *I*_2D_/*I*_G_ ratio has a maximum approximately equal to one for the Raman data with the 2D peak FWHM smaller than 50 cm^−1^ (Figure 7e). Domains of sample A that correspond to this set of Raman data can be attributed to a single or double layer of graphene. According to Costa et al., a single layer of graphene on a SiO_2_ substrate with a laser excitation wavelength of 473 nm should exhibit the 2D band position at 2703 cm^−1^[24]. The significant blue shift for the 2D band position (2718 cm^−1^) could be caused by a double layer structure of graphene [26] and/or a doping effect [16]. The G-band position at 1590 cm^−1^ (Figure 7a) is also blue-shifted with respect to the accepted standard value of 1580 cm^−1^ for the undoped graphene [25]. Further, Raman data with the 2D peak FWHM greater than 50 cm^−1^ (Figure 7b,d,f,h) also exhibit rather monotonic distribution of values with a single maximum. The G-band position together with the blue-shifted 2D position and a sharp peak at 0.3 for the *I*_2D_/*I*_G_ ratio allow the association of these domains of sample A with a larger thickness. Indeed, the significant difference of the G band intensity between these two sets (Figure 7g and Figure 7h) confirms this statement.

**Figure 8:**
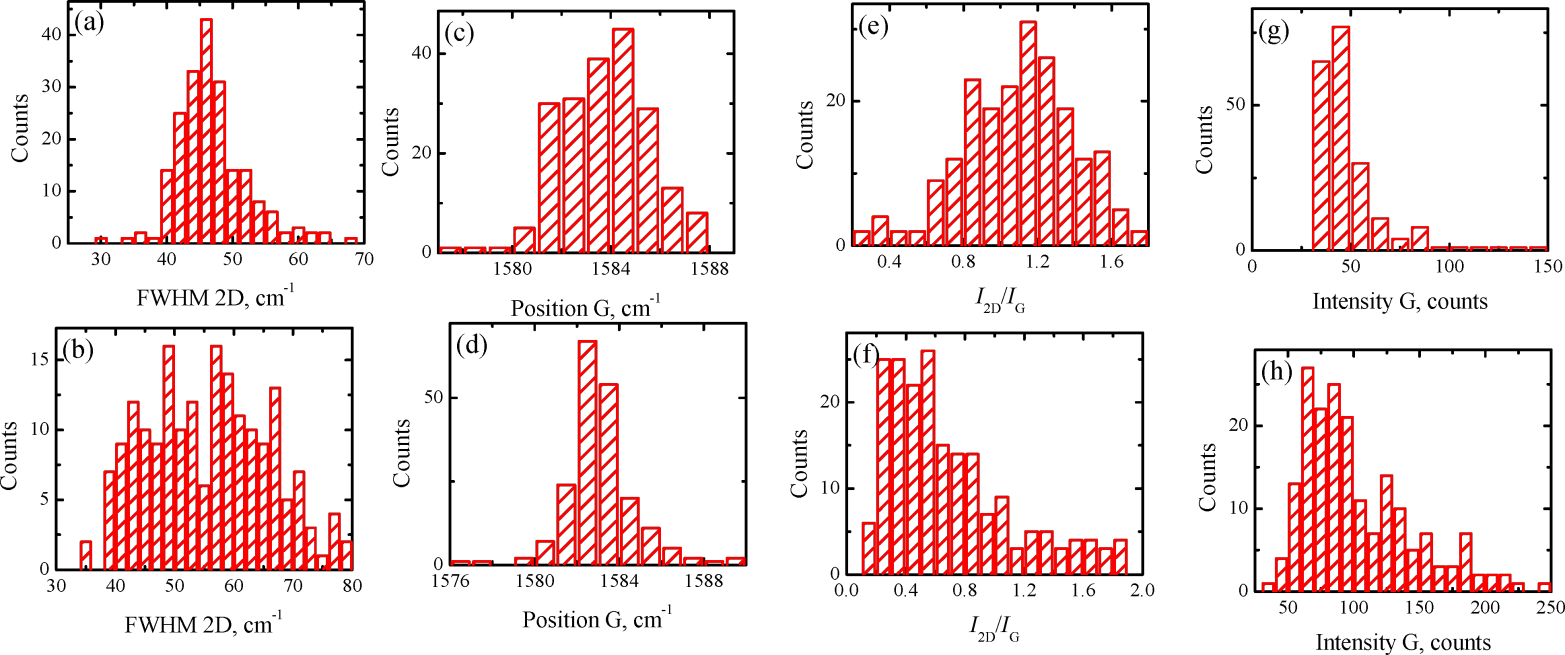
Replotted histograms of sample B on a SiO_2_/Si substrate for a laser excitation wavelength of 473 nm using the 2D-band position of 2712 cm^−1^ as the partitioning criterion. (a) and (b) FWHM of the 2D band. (c) and (d) G-band position. (e) and (f) *I*_2D_/*I*_G_ ratio. (g) and (h) *I*_G_ value. Data shown in (a), (c), (e) and (g) plots are for the 2D-band positions greater than 2712 cm^−1^. Data shown in (b), (d), (f) and (h) plots are for the 2D-band positions smaller than 2712 cm^−1^.

As we mentioned above, the non-monotonic distribution is a characteristic feature of the obtained histograms. Thus, the partitioning of the Raman data set could be based on different parameters, *I*_2D_/*I*_G_, FWHM of the 2D peak, or the position of the 2D or G band. For both samples, we performed the partitioning procedure following different parameters (this result is not shown here). The obtained results are in reasonable agreement with each other. Therefore, in order to demonstrate the universality of the elaborated method, for sample B on a SiO_2_/Si substrate, the Raman data set splitting was based on another criterion, namely, the 2D-band position with a splitting value of 2712 cm^−1^ (Figure 8a–h). This was used with the same laser excitation wavelength as for sample A (473 nm).

Data for the 2D-band positions greater than 2712 cm^−1^ are presented in Figure 8a,c,e,g, while data for the 2D-band positions smaller than 2712 cm^−1^ are shown in Figure 8b,d,f,h. By analogy with the previous results, the larger *I*_2D_/*I*_G_ ratio for the G-band position greater than 2712 cm^−1^ (Figure 8e and Figure 8f) and two times difference in the *I*_G_ values (Figure 8g and Figure 8h) allow for the association of domains with the 2D band position smaller than 2712 cm^−1^ with SLG [30].

### XPS results

We now compare the parameters of Raman spectra which we attribute to the SLG domains in samples A and B (473 nm excitation wavelength). The maxima of the G-band position distribution for the SLG fraction of sample A and B are centered at ≈1590 cm^−1^ (Figure 7a) and ≈1585 cm^−1^ (Figure 8c), respectively. The maxima of the *I*_2D_/*I*_G_ ratio are centered at ≈1 (Figure 7e) and ≈1.2 (Figure 8e) for samples A and B, respectively. Both of these facts indicate that sample A has a higher carrier concentration with respect to sample B [27,31]. Considering that for both samples the substrate material, transfer method and storage conditions were the same, it is reasonable to suppose that the main source of the change may originate from doping during the growth. Since we use nitrogen as a carrier gas, the probability of this scenario could be high. Actually, XPS results can give useful qualitative and quantitative information about graphene doping. Thus, to check the importance of nitrogen doping we performed an XPS study for both samples.

**Figure 9:**
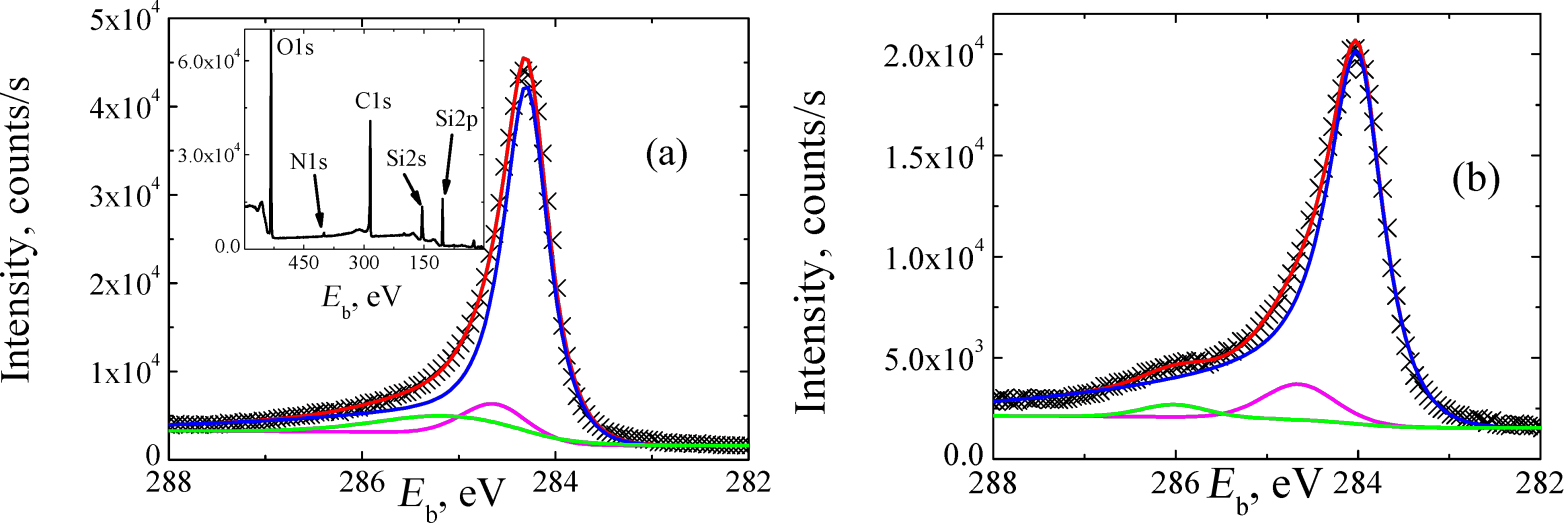
High-resolution C 1s XPS spectra of samples on a SiO_2_/Si substrate. (a) Raw data sample A (black crosses). Blue, magenta and green lines are the result of the fitting procedure. The red line corresponds to the envelope of the fitted peaks. Inset: XPS survey spectrum. (b) Raw data sample B (black crosses). Blue, magenta and green lines are the result of the fitting procedure and the red line corresponds to the envelope of the fitted peaks.

In the inset of Figure 9a we show the XPS survey spectrum of sample A transferred onto a SiO_2_/Si substrate. The main core level peaks for carbon, nitrogen, silicon and oxygen are indicated. It is important to note that due to the small thickness of the samples, the contribution from the substrate in the form of strong silicon and oxygen signals was detected. The presence of the nitrogen 1s core level in the XPS survey spectrum could be caused either by adsorption or/and incorporation of nitrogen into graphene with the formation of C–N bonds. These bonds affect the shape and position of the carbon 1s response. Figure 9a and Figure 9b present the C1s core level for samples A and B, correspondingly. The experimental data could be deconvoluted into three peaks. The first, dominant peak, indicated by a blue line, has a maximum position of 284.3 eV and 284.08 eV and FWHM of 0.54 eV and 0.68 eV for samples A and B, respectively. We attribute this line to the sp^2^C bonds. The dominance of this peak confirms the fact that most of the carbon atoms are arranged into a honeycomb lattice. The sp^2^C peak of graphene could be observed at various values of energy depending on the material of the substrate on which graphene was deposited. The peak position varies from 283.97 eV for graphene on Pt(111) [32–33] to 284.83 eV for graphene on SiC [34–36]. This variation of the sp^2^C peak position is usually explained by the charge transfer phenomena that take place in the substrate–graphene system [37]. Since both samples were deposited on the same substrate, we expect the same impact of the charge transfer on the peak position. The difference in the binding energy *E*_b_ can be related to the difference in concentration of sp^2^C–H bonds which cause the down shift of the *E*_b_[38]. The origin of such bonds and its impact on the graphene growth will be discussed further in the text. The second peak (a magenta line) of the C1s core level, centered at 284.63 eV and 284.65 eV for samples A and B, respectively, is slightly up-shifted compare to the dominant one, and could be attributed to the existence of sp^3^C bonds [39–40]. Finally, the third peak, indicated by a green line, with the characteristic energy of 285.12 eV and 286.02 eV for samples A and B, respectively, can be attributed to the presence of sp^2^C–N bonds [41–44]. The performed analysis of the C1s core level data confirms the fact that the interpretation of the C1s data for graphene is not a trivial task [37]. A number of factors, such as oxygen-based functional groups, charge compensation effects and doping can significantly affect the C 1s response [37–38]. Therefore, analysis of the N 1s core level is necessary to verify the status of nitrogen.

**Figure 10:**
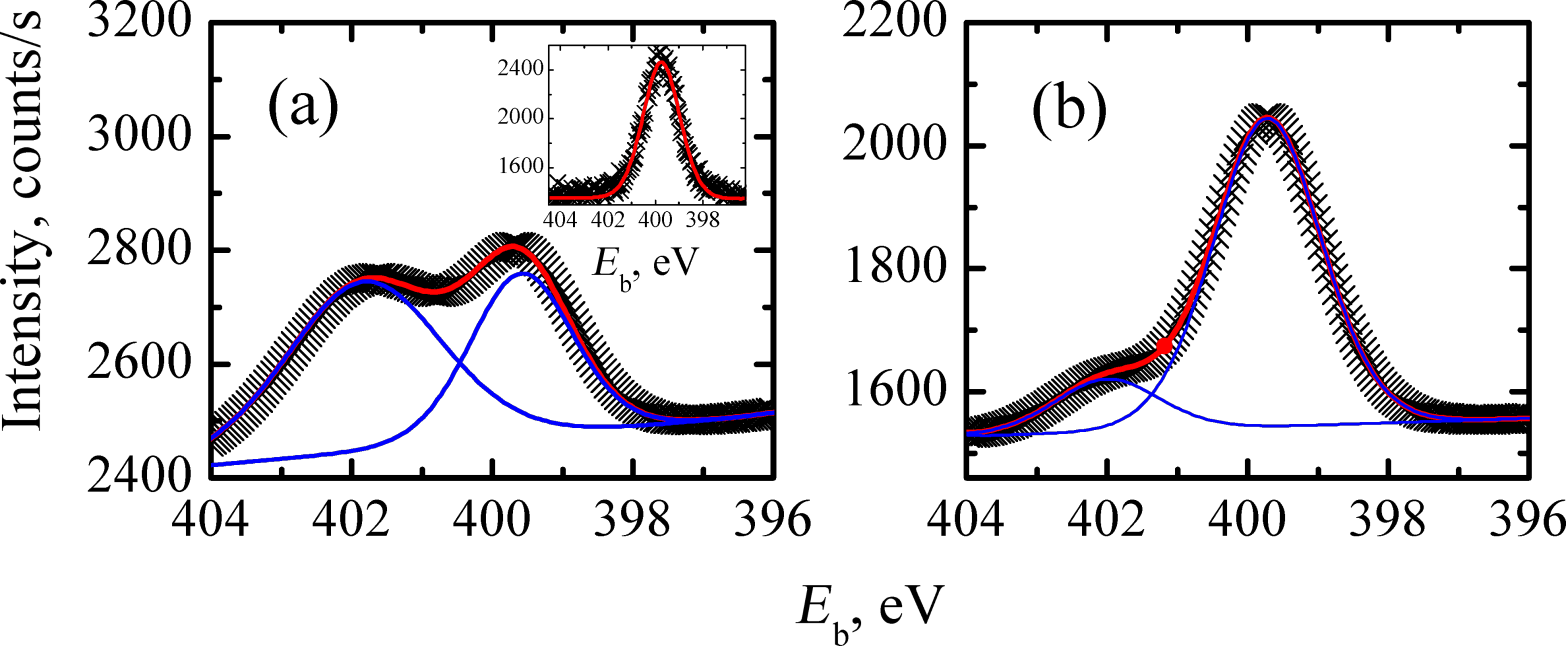
High-resolution N 1s XPS spectra of samples on SiO_2_/Si substrate. (a) Raw data for sample A (black crosses). The blue line is the result of the fitting procedure and the red line corresponds to the envelope of the fitted peaks. Inset: N 1s XPS spectrum of a bare substrate (black symbols) together with the fitting curve (red). (b) Raw data for sample B (black crosses). The blue lines are the result of the fitting procedure and the red line corresponds to the envelope of the fitted peaks.

Figure 10a and Figure 10b present the high-resolution XPS data for the N 1s spectrum of samples A and B, respectively, in which the spectra could be fitted to two components of the binding energy, namely at *E*_b_ = 399.7 eV and 402.1 eV (sample B) (*E*_b_ = 401.8 eV (sample A)). The peak at *E*_b_ = 399.7 eV observed for both samples is related to the adsorbed nitrogen. Its position was quantified by the measurement on the bare substrate area, see inset of Figure 10a, indicating that nitrogen dopes both samples with a single status. It is rather difficult to associate the binding energy with a specific configuration of nitrogen and this usually requires additional structural measurements. Indeed, the energy range 398–404 eV can be related to the different nitrogen configurations, such as pyridine, pyrrolic, graphite, and pyridine N-oxide (see, e.g., [45]). We also cannot exclude the formation of a very stable nitrile CN bond (sp configuration) [46]. Based only on the XPS data we cannot make an unambiguous assignment concerning the nitrogen configuration. For example, the binding energy for graphitic nitrogen is varied between 400.0 eV [47] and 402.7 eV [44]. However, two solid conclusions can be made from the obtained XPS data: (i) nitrogen is incorporated into the graphene in a single status; (ii) the concentration of nitrogen associated with the energy of about *E*_b_ ≈ 402 eV is greater for sample A. We would like to emphasize that these observations are consistent with the Raman spectroscopy results, from which it follows that the G-band blue shift is greater for sample A.

Finally, the quantified surface atomic concentrations determined by XPS for samples A and B are summarized in Table 1.

**Table 1:** Quantified surface atomic concentrations for samples A and B on SiO_2_/Si substrate as obtained from the XPS study.

Sample	Atomic concentration, %		
	C	N (399.74 eV)	N (≈402 eV)

A	98.4	0.6	1.00
B	97.6	1.99	0.41

### Comparative analysis of the defect concentration from XPS and Raman data

The XPS measurements provide the atomic concentration of nitrogen in the studied samples, as shown in Table 1. For further discussion, it is more suitable to convert atomic concentration to the surface concentration. For sample B the nitrogen atomic concentration is ≈0.4%, meaning there is about one nitrogen atom per 250 carbon atoms. Taking into account the surface concentration of carbon atoms in graphene, ≈3.8 × 10^15^ cm^−2^, the average surface concentration of the nitrogen atoms in sample B can be evaluated as *n*_N_ ≈ 1.5 × 10^13^ cm^−2^.

On the other hand, the Raman integral intensity ratio *I*_D_/*I*_G_ provides information about the concentration of point defects *n* [29],

[1]
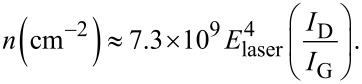


From the experimental results, *I*_D_/*I*_G_ ≈ 1, and the energy of the laser *E*_laser_ ≈ 2.33 eV, we get *n* ≈ 2.2 × 10^11^ cm^−2^.

The reason for lower defect density as compared to the *n*_N_ value may be the following. The nitrogen atoms could be located in such a configuration that almost does not affect the *I*_D_ value. In particular, the pyrrolic nitrogen due to the symmetry breaking of hexagon rings should have a strong impact on the intensity of the D band and cannot be considered as the main type of defect in the studied films.

### Twisted graphene

**Figure 11:**
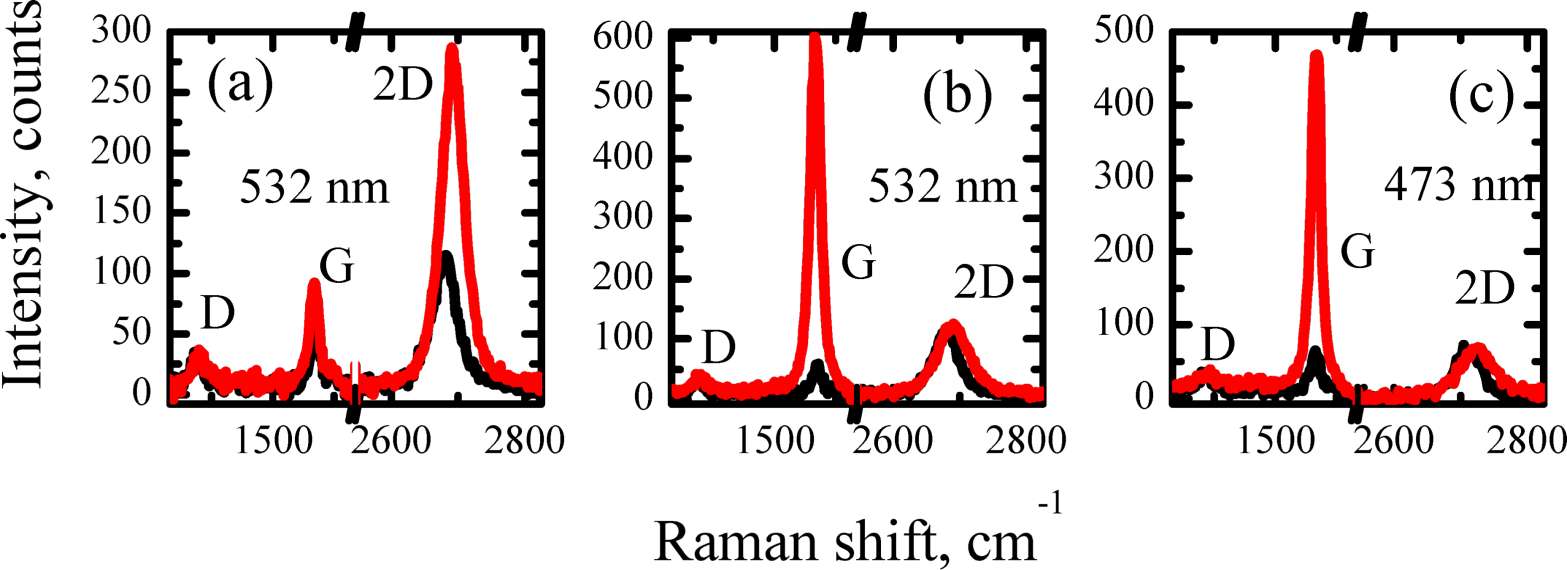
Raman spectra of sample B on SiO_2_/Si substrate. (a) Raman spectra of SLG (black) and double layer graphene (red) with the laser excitation wavelength of 532 nm. (b) Raman spectra of SLG (black) and double layer graphene with the G resonance (red) with the laser excitation wavelength of 532 nm. (c) Raman spectra of SLG (black) and double layer graphene with the G resonance (red) with the laser excitation wavelength of 473 nm.

Now we focus on the Raman data associated with the double layer graphene. In Figure 11a we plot selected spectra associated with the SLG (black line) and double layer (red line) sets. An almost three times higher 2D-band intensity and its blue shift as compared with the SGL spectrum indicates the twisted nature of the double layer graphene in sample B. Moreover, as we pointed out in the Introduction, one of the most attractive feature of the TG electronic structure is presence of the vHs. In Raman spectroscopy, the presence of the vHs yields the G-resonance [48]. It consists of a more than one order of magnitude enhancement of the G-band intensity when the excitation energy fits the vHs energy difference. Indeed, the domains with a G intensity an order of magnitude higher with respect to that of the SGL were observed for both laser excitation wavelengths, 532 nm (Figure 11b) and 473 nm (Figure 11c).

For the domains corresponding to the spectra presented in Figure 11b and Figure 11c it is possible to evaluate the rotation angle θ. For small θ values [48]

[2]
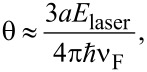


where *a* is the lattice parameter of graphene (2.46 Å), 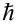 is the reduced Planck’s constant, *v*_F_ is the Fermi velocity in monolayer graphene (10^6^ m/s) and *E*_laser_ is the laser energy. Within this approach we obtain θ = 13.4° and 11.9° for the laser excitation wavelengths of 473 and 532 nm, respectively. The estimated θ values mean that in the investigated domains, the layers of graphene could be electronically decoupled [4–5].

The higher G band intensities were observed at different locations within the same probed area for different excitation wavelengths. This fact confirms that the presence of the G-resonance is a consequence of the twisted nature of the graphene. However, the ratio of integrated G-band intensities for the double layer TG and SGL is ≈60 and ≈30 for excitation wavelengths of 532 and 473 nm, respectively [48]. In our case, it is close to 10 for both wavelengths. Such a difference can be reasonably explained by the presence of nitrogen in the graphene structure (as confirmed by XPS) and the accompanying stress or polycrystalline nature of the studied thin film. Actually, both factors lead to the decrease of the ratio intensity.

It is worth highlighting that the above presented Raman mapping has been performed on different sections of samples (not shown here). One of the main results of this study is the uniformity of the samples, confirming their macroscopic homogeneity. For sample B we have provided sets of data where SLG fraction was significant (≈50%), which helps to evaluate and compare the structural properties of SLG and TG. However, for other sets of data, the fraction of double layer TG dominates.

**Figure 12:**
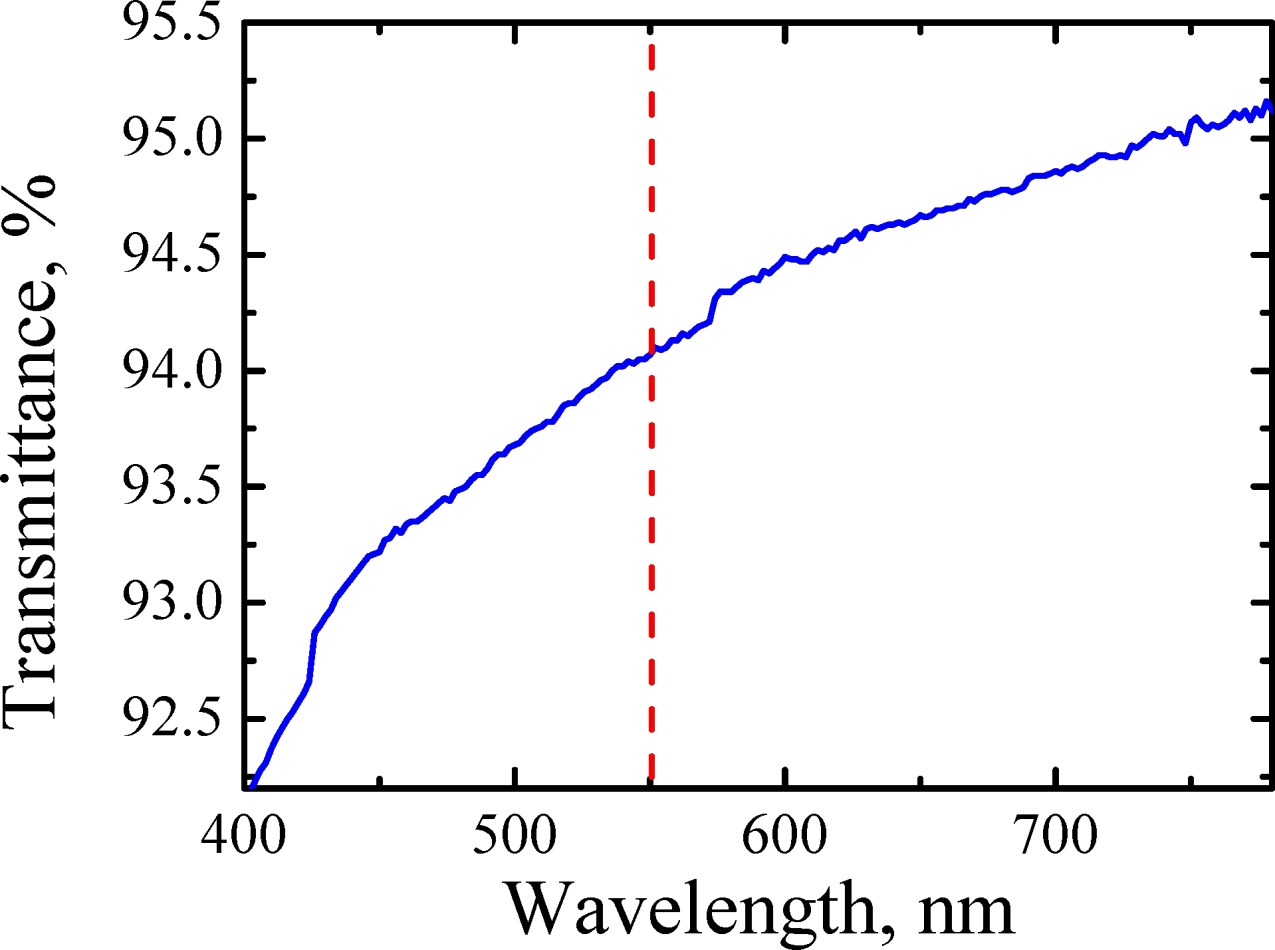
The transmittance of sample B versus wavelength on a glass substrate. The vertical dashed line indicates the wavelength at which the transmittance was estimated.

We also proved homogeneity directly by the light transmittance measurement of samples transferred on a glass substrate. The diameter of probed area was ≈0.5 cm. In Figure 12 we show the transmittance for sample B recorded in the 400–800 nm range. It follows that the transmittance at 550 nm is 94%. This value corresponds to a number of graphene layers between 2 (95.5%) and 3 (93.3%) [26]. It is worth mentioning that the twisted nature of graphene slightly increases the absorption of light [49–50]. This fact could be the reason for the slight discrepancy found while evaluating the number of layers from the results of Figure 12.

### Impact of hydrogen flow on graphene structural properties

Now we would like to discuss the two most prominent phenomena observed in our experiments: (i) the morphology change of the films with hydrogen flow rate and (ii) nitrogen atom incorporation into graphene, which we believe to be correlated phenomena. Nevertheless, as a first approach, we will discuss them separately.

Let us underline the main features that distinguish the morphology of studied samples. Sample A is characterized by the presence of hexagonally shaped domains with a few and single layer graphene domains. In turn, sample B contains the mixture of single and double layer domains where the double layer fraction dominates. Our studies do not indicate the presence of any regularity in shape and distribution of SLG and double layer domains at the measured scale. Since the hydrogen flow rate value was the only technological parameter we have varied during the synthesis, we will focus on the impact of hydrogen on the graphene. First of all, the hydrogen partial pressure strongly affects the shape of the graphene edges. It was demonstrated that higher hydrogen pressure favors the hexagonal shape whereas low hydrogen pressure makes dendritic-like growth preferable [51–52]. Our observations, pointed out above, are consistent with these findings.

The formation of few layer graphene requires formation of the additional graphene layer (layers) on the top or underneath of a SLG. These processes are usually described by wedding cake (WC) [53–54] and inverse wedding cake (IWC) [55–56] models, respectively. Detailed experimental studies strongly support the IWC model of growth [55–57]. It was theoretically shown [58] that the diffusion of C adatoms underneath an existing graphene top layer (GTL) is much faster than on a Cu surface free of graphene. Moreover, the hydrogen pressure plays a crucial role in the growth activity of graphene edges [58]. In particular, the low pressure of hydrogen yields the passivation of graphene edges by copper, which prevents the diffusion of C adatoms underneath the GTL and thus contributes to the formation of SLG. Conversely, at high hydrogen pressure, the graphene edges are terminated by hydrogen atoms, which inhibits the carbon adsorption and few layer graphene growth is favored [58]. Taking into account that double and few layer graphene is really synthesized under our experimental conditions, it is possible to conclude that the partial pressure of hydrogen is still above the threshold of H-termination for a copper catalyst. In addition, as it was shown in [59] for dendritically shaped graphene single crystals (low partial pressure of hydrogen), multiple small adlayers are favorable. Therefore, we believe that during the C_10_H_22_ decomposition there is enough hydrogen to terminate the graphene edges [13].

Finally, we turn to the problem of nitrogen incorporation into graphene. Nitrogen can be incorporated into graphene sheet (i) in situ, using ammonia as a component of the gas carrier mixture [43] or with nitrogen containing precursors [60–61] and (ii) by post-treatment, e.g., by treatment in ammonia plasma [62] or N-ion irradiation [63]. To the best of our knowledge, there is only one article where N_2_ gas was used as the nitrogen source during the CVD growth [64]. In our opinion, the main difficulties in using N_2_ gas as a nitrogen source arises from the fact that nitrogen molecule possesses one of the strongest bonds with an energy of 226 kcal/mol, which means that the temperature of the CVD process is not enough to decompose a significant amount of nitrogen molecules into atoms. The authors of the work [64] did not explore the mechanism of nitrogen incorporation. We believe that in our case the efficient decomposition of nitrogen occurs due to the presence of hydrocarbon in the reactive mixture. Indeed, as it was shown by C. P. Fenimore, carbon (^•^C) or hydrocarbon (^•^C_x_H_y_) radicals may attack on nitrogen molecules [65]. The endothermicity of such a reaction can be an order of magnitude smaller than for a N_2_ dissociation process. For example, for the ^•^CH + N_2_ = N + ^•^CHN reaction this value was determined as 21.2 ± 0.7 kcal/mol [66]. Moreover, as it was proved by time-of-flight mass spectrometry experiments, various hydrocarbon radicals can be formed from *n*-decane just by thermal decomposition [67]


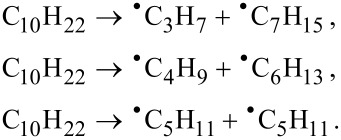


However, the issue of the exact reaction route of *n*-decane decomposition at elevated temperature over copper catalyst is beyond the scope of our study. We can only reasonably assume that the different partial pressure of H_2_ in a gas mixture could favor some decomposition path. As a consequence, it enhances the concentration of radicals with specific configurations. Obviously, the enthalpy of the hydrocarbon reaction with N_2_ depends on the hydrocarbon radical configuration [68]. Therefore, using these arguments the variation in doping level for samples A and B could be tentatively explained.

## Conclusion

In summary, the graphene films have been studied by using the micro-Raman technique. The samples have been grown on copper foil by APCVD using n-decane as a precursor and a mixture of nitrogen and hydrogen as a carrier gas with the use of different hydrogen flow rates. A special analysis approach to the Raman data was employed based on the statistical analysis of spectral line parameters. This approach allowed the association of Raman spectra to fractions of the films with different thicknesses. Based on the values of the 2D peak FWHM and 2D band position, the double layer fraction of graphene grown with the lower hydrogen feeding rate has been established. Moreover, the analysis of the Raman spectra revealed the presence of graphene spots with the G-resonance for both excitation wavelengths used in our experiments (473 nm and 532 nm). The observation of the G-resonance directly confirms the twisted nature of graphene. The obtained blue shift of the G and 2D band positions of the SLG fractions is caused by nitrogen doping, which has been proved by the XPS study. The binding energy of incorporated nitrogen has been evaluated to be around 402 eV. The amount of the G-band shift for each sample is consistent with the XPS data. It has been found that at the wavelength of 550 nm the transmittance for the film grown with the lower hydrogen feeding rate is equal to 94%, which corresponds to 2–3 graphene layers. This is in good agreement with the micro-Raman findings. We suppose that the variation in the morphology is presumably related to the variation of the hydrogen flow in our experiments, as it has been also demonstrated by other authors. Finally, the possible mechanism of the nitrogen concentration incorporated in graphene based on a variation of the endothermicity of a ^•^C_x_H_y_ + N_2_ reaction has been proposed.

Further work related to the mechanisms of the nitrogen doping and relation between transport properties of graphene and its microstructure is in progress now.
